# The *Campylobacter jejuni* Response Regulator and Cyclic-Di-GMP Binding CbrR Is a Novel Regulator of Flagellar Motility

**DOI:** 10.3390/microorganisms10010086

**Published:** 2021-12-31

**Authors:** Claudia A. Cox, Marek Bogacz, Faiha M. El Abbar, Darren D. Browning, Brian Y. Hsueh, Chris M. Waters, Vincent T. Lee, Stuart A. Thompson

**Affiliations:** 1 Department of Medicine, Division of Infectious Diseases, Augusta University, Augusta, GA 30912, USA; cfulmer@augusta.edu (C.A.C.); mbogacz@emory.edu (M.B.); felabbar@uottawa.ca (F.M.E.A.); 2 Department of Biochemistry and Molecular Biology, Augusta University, Augusta, GA 30912, USA; dbrowning@augusta.edu; 3 Department of Microbiology and Molecular Genetics, Michigan State University, East Lansing, MI 48824, USA; hsuehbri@msu.edu (B.Y.H.); watersc3@msu.edu (C.M.W.); 4 Department of Cell Biology and Molecular Genetics, University of Maryland, College Park, MD 20742, USA; vtlee@umd.edu

**Keywords:** flagella, motility, c-di-GMP, regulation, pathogenesis, biofilm

## Abstract

A leading cause of bacterial gastroenteritis, *Campylobacter jejuni* is also associated with broad sequelae, including extragastrointestinal conditions such as reactive arthritis and Guillain-Barré Syndrome (GBS). CbrR is a *C. jejuni* response regulator that is annotated as a diguanylate cyclase (DGC), an enzyme that catalyzes the synthesis of c-di-GMP, a universal bacterial second messenger, from GTP. In *C. jejuni* DRH212, we constructed an unmarked deletion mutant, *cbrR^−^*, and complemented mutant, *cbrR^+^*. Motility assays indicated a hyper-motile phenotype associated with *cbrR^−^*, whereas motility was deficient in *cbrR^+^*. The overexpression of CbrR in *cbrR*^+^ was accompanied by a reduction in expression of FlaA, the major flagellin. Biofilm assays and scanning electron microscopy demonstrated similarities between DRH212 and *cbrR^−^*; however, *cbrR^+^* was unable to form significant biofilms. Transmission electron microscopy showed similar cell morphology between the three strains; however, *cbrR^+^* cells lacked flagella. Differential radial capillary action of ligand assays (DRaCALA) showed that CbrR binds GTP and c-di-GMP. Liquid chromatography tandem mass spectrometry detected low levels of c-di-GMP in *C. jejuni* and in *E. coli* expressing CbrR. CbrR is therefore a negative regulator of FlaA expression and motility, a critical virulence factor in *C. jejuni* pathogenesis.

## 1. Introduction

As the leading cause of foodborne illness tracked by the Foodborne Diseases Active Surveillance Network (FoodNet) in the United States in 2016–2019 [1], *Campylobacter jejuni* causes 450 million annual cases of bacterial gastroenteritis worldwide [2] and approximately 40,000 deaths each year in children aged five and younger [3]. Infections are most often attributed to the consumption of undercooked meat, especially poultry [4,5,6,7], but disease also results from consumption of raw or contaminated milk [8] or from exposure to environmental sources such as surface water [9,10]. Campylobacteriosis most often presents as a week-long course of bloody diarrhea, fever, vomiting, headache, nausea, and intense abdominal pain [5] and is endemic in portions of Africa, the Middle East, and Asia [4]. Infection does not lead to lifelong immunity against the bacteria; therefore, individuals are left at risk of subsequent *Campylobacter* infection [11]. Further, gastrointestinal symptoms are not the only threat associated with *Campylobacter*, as several peripheral neuropathies including Guillain–Barré syndrome (GBS) have been linked to the bacteria [4,12,13]. While the *Campylobacter* genus has been recognized since 1963 [4], there is no vaccine nor prophylactic available to prevent *Campylobacter* infections [12,14] and its pathogenic mechanisms remain enigmatic.

*C. jejuni* is able to vary its gene expression in response to diverse in vivo and environmental cues; however, it does not contain nearly the number of regulatory proteins that many other bacteria do. *Escherichia coli* has seven different sigma factors and approximately 300 transcription factors for its ~4500 genes [15,16]. Compared to the three sigma factors and 34 transcriptional regulators found in *C. jejuni* for its ~1700 genes, it becomes clear that survival, environmental responses, and virulence are all controlled by a very small portion of its proteins (approximately 2% of its proteome) [17]. CbrR (*Campylobacter* bile resistance regulator) [18], one of the putative transcriptional regulators in *C. jejuni*, is an orphan response regulator with a predicted, but highly variant, GGDEF domain, indicative of diguanylate cyclase (DGC) activity.

DGCs are enzymes that catalyze the hydrolysis of two molecules of guanosine triphosphate (GTP) to form cyclic diguanylate monophosphate (c-di-GMP), a small molecule often used as a second messenger in bacteria [19]. DGCs are often activated by environmental signals that indicate a phenotypic shift from a motile to sessile cell type [20,21], while also commencing biofilm formation, the appearance of small colony variants, the secretion of extracellular polymeric substances, and a decrease in virulence [19,22]. A reversion to a motile, virulent phenotype can be induced by the degradation of c-di-GMP by phosphodiesterases (PDEs) that contain either EAL or HD-GYP domains [22]. Targets for c-di-GMP binding include riboswitches, short mRNA structures able to bind specific small molecules that regulate gene expression [23], transcription factors necessary to activate genes that affect the phenotypic changes associated with c-di-GMP production [24], and proteins that contain PilZ domains, which have various functions (cellulose synthesis, alginate biosynthesis, motility reduction), but more than half the proteins that possess PilZ domains have unknown functions [25].

CbrR has been partially characterized in *C. jejuni*, as a *C. jejuni cbrR* mutant showed reduced bile resistance and a decrease in chick colonization [18]. It has also been shown that this gene is downregulated during chick colonization, when compared to the inoculum [26,27]. Organisms that utilize c-di-GMP produced by DGCs and degraded by PDEs typically encode several of these proteins within their genomes [28]; however, *C. jejuni* possesses only the single annotated DGC in CbrR, and no PDEs or c-di-GMP receptors have yet been identified. Riboswitches are poorly characterized in *C. jejuni* [26], and PilZ domain proteins have also not yet been identified in *C. jejuni*. *C. jejuni* CbrR interacts with the chemotaxis protein CheA, suggesting that CheA may be involved in regulating CbrR activity via phosphorylation of CbrR receiver domains [29].

Motility provided by polar flagella is a known critical virulence factor of *C. jejuni*, with nonmotile strains showing attenuated mouse and chick colonization [30,31,32,33]. The importance of flagellar motility has also been indicated in human cases of campylobacteriosis [34], including the role that flagella play in the interaction between *C. jejuni* and host cells [35,36,37]. As *C. jejuni* must travel through the viscous mucus layer protecting the intestinal epithelial cells, motility is a necessary phenotype to ensure interaction between bacteria and host cells [7,38,39]. Those *Campylobacter* strains shown to exhibit this interaction, in the forms of adherence and invasion of host cells, have been shown to be associated with increased pathogenicity [40].

The ability of *C. jejuni* to form biofilm has been suggested to be crucial to the persistence in the environment of this microaerophilic microbe [41]. This may be due to the fact that the polymeric matrix that comprises the biofilm; composed of extracellular polymeric substances (EPS) such as DNA, proteins, and/or polysaccharides [42]; serves to protect the bacteria from desiccation [43], antibiotics [44], disinfectants [43], and host immune responses [44]. While *C. jejuni* biofilms have not yet been found in human infections, these bacteria are able to form these protective matrices in the environment [45], on chicken skin [43], on ex vivo human intestinal tissue [46], and both flagella and flagella-mediated motility are involved [47,48,49]. This is likely due to the role of autoagglutination (AAG), the tendency of cells to aggregate via their heavily glycosylated flagella [39], giving rise to microcolonies which are the initial step of biofilm formation [39,50].

As *C. jejuni* CbrR is as of yet incompletely characterized, our goal for this study was to understand the mechanisms by which this putative c-di-GMP-related regulatory protein functions. While annotated as a DGC, synthesis of c-di-GMP in *C. jejuni* has not previously been shown, nor has a possible mechanism of activation of CbrR. However, we now show that CbrR binds c-di-GMP and provide evidence to support a role for CbrR in regulating *C. jejuni* flagellar motility. Together, this provides evidence of a novel c-di-GMP system in *C. jejuni*, with a predicted role in pathogenesis.

## 2. Materials and Methods

### 2.1. Bacterial Strains and Routine Growth Conditions

Bacterial strains used in this study are listed in Supplementary Table S1. Primers used are available in Supplementary Table S2. All *C. jejuni* experiments used DRH212, a streptomycin-resistant derivative of 81–176, (kind gift of David Hendrixson) as the wild type (WT). WT, *cbrR^−^*, and *cbrR^+^* complemented mutant were cultured on Mueller–Hinton (MH) agar plates or in biphasic cultures constituted of a layer of MH agar overlaid with 25 mL MH broth in a 75 cm^2^ tissue culture flask, supplemented with streptomycin (2 mg/mL) or chloramphenicol (10 μg/mL) when appropriate, and incubated at 42 °C either in a tri-gas incubator (10% CO_2_, 10% O_2_, 80% N_2_) or in Mitsubishi AnaeroPack® boxes using AnaeroPack®-MicroAero (Mitsubishi Gas Chemical America, New York, NY, USA) sachets to generate microaerophilic conditions (6–10% O_2_, 5–8% CO_2_). *E. coli* strains were grown in LB broth or agar supplemented with ampicillin (100 μg/mL) and/or chloramphenicol (30 μg/mL) when appropriate. *E. coli* One Shot™ TOP10 chemically competent cells (Thermo Fisher, Waltham, MA, USA) were used for plasmid cloning, *E. coli* C2987 chemically competent cells were used for site-directed mutagenesis experiments, and *E. coli* BL21(DE3) chemically competent cells were used for protein expression experiments. *E. coli* cultures were incubated at 37 °C. All strains were stored at −80 °C in LB broth with 35% (*v*/*v*) glycerol.

### 2.2. cbrR^−^ Mutant Construction

To create an unmarked deletion of the *cbrR* gene within *C. jejuni* DRH212 cells, a streptomycin counterselection technique was used, as described [51]. To achieve this, the *cbrR* gene was amplified using primers designed using the National Center for Biotechnology Information (NCBI) database sequence from accession number CP000538.1 locus tag CJJ81176_0671 (base pairs 598708-599952), which includes a 500 base-pair flanking region on either side of the *cbrR* coding sequence (CDS) for homologous recombination into the DRH212 chromosome. PCR was performed using 81–176 chromosomal DNA with primers CF101 and CF102 and cloned into pCRII-TOPO (Thermo Fisher, Waltham, MA, USA) to make pCAF101. Inverse PCR was then performed on pCAF101 to remove 97% of the *cbrR* CDS, but keep the remaining *cbrR* sequence in-frame, and retain the ribosome binding site for the proximal downstream gene to help ensure that transcription of genes downstream would be unaffected [52]. The resulting PCR product using primers CF103 and CF104, containing AgeI (both primers) and NheI (CF104) restriction sites was digested with AgeI and self-ligated to create pCAF102. Primers *rpsL_HP_*-F1 and CF105 were used to amplify the *rpsL_HF_-cat* antibiotic cassette from pKR021 [51] and were cloned into pCRII-TOPO to create pCAF103. Afterwards, pCAF103 was digested with AgeI and NheI (New England Biolabs, Ipswitch, MA, USA) to release the antibiotic cassette which was gel purified (Qiagen, Hilden, DE) and ligated into AgeI- and NheI-digested pCAF102 to create pCAF104. DRH212 cells were made electrocompetent as previously described [53] using 10% ice-cold glycerol as electroporation buffer and resuspended in 40 µL ice-cold GYT medium (10% (*v*/*v*) glycerol, 0.125% (*w*/*v*) yeast extract, 0.25% (*w*/*v*) tryptone). Competent DRH212 cells were then electroporated with pCAF104 and resulting Str^S^/Cm^R^ colonies were screened using PCR primers CF101 and CF102 to confirm successful replacement of the *cbrR* CDS with *rpsL_HF_-cat*. These intermediate mutant *C. jejuni* colonies were then immediately electroporated with pCAF102 to replace the antibiotic resistance cassette and resulting Str^R^/Cm^S^ colonies were screened using PCR primers CF101 and CF102 to confirm successful unmarked deletion of the *cbrR* gene.

### 2.3. Complementation of cbrR^−^ Mutant

To reintroduce the *cbrR* gene within the intergenic space between genes encoding the rRNA subunit 16S and the tRNA-Ala molecule in the *cbrR^−^* mutant chromosome, primers CF110 and CF111 were used to amplify the *cbrR* sequence from pCAF101 adding XbaI sites and cloned into pRRC [54] to yield pCAF108. Natural transformation [55] was performed on agar-grown cells which were incubated for two hours (h) on non-selective MH agar, then 5 μg pCAF108 (or phosphate-buffered saline (PBS) for negative control) was added and incubated for four h. Cells were then harvested, suspended in PBS, and plated onto MH Cm agar plates. Colonies were picked and screened using primers CF110 and CF111 to indicate successful reintroduction of *cbrR*.

### 2.4. Purification of CbrR Protein

To express a His-tagged recombinant protein, *cbrR* was amplified from pCAF101 using primers CF112 and CF113 and ligated into pET-20b(+) to add a C-terminal His_6_-tag for purification purposes, creating pCAF107. Protein was affinity-purified by binding to Ni-NTA agarose (Thermo Scientific, Waltham, MA, USA) and subsequently further purified via ion-exchange chromatography on a Mono Q (Cytiva, Marlborough, MA, USA) column (binding buffer: 20 mM Tris-HCL, pH7.5; 1 mM DTT; elution buffer: 20 mM Tris-HCL, pH7.5; 1 mM DTT; 1 M NaCl) and dialyzed into PBS for polyclonal rabbit antiserum generation (Cocalico Biologicals, Stevens, PA, USA) (Supplementary Figure S1). Western blots were performed on 1 μg total protein from whole-cell lysates of all three strains using antisera against CbrR, FlaA (each at 1:4000 dilution) or FliF (at 1:2000 dilution) as primary antibodies, and goat anti-rabbit HRP-conjugate IgG (1:20,000) or goat anti-mouse HRP-conjugate IgG (1:100,000) as secondary antibodies. SuperSignal™ West Pico PLUS Chemiluminescent Substrate (Thermo Scientific, Waltham, MA, USA) was used to develop Western blot images. RNA-Seq experiments (manuscript in preparation) show that FliF is expressed equally in these strains; therefore, anti-FliF antibodies were used to demonstrate equivalent loading of the protein samples.

### 2.5. Motility Assays

*C. jejuni* cells harvested from overnight agar plates were suspended or grown in MH broth to an optical density at 600 nm (OD_600_) of 0.1. Subsequently, 2 μL of each suspension was used to inoculate the soft agar plates (0.4% agar) approximately halfway through the agar to ensure cells would be travelling through the agar and not swarming on the surface. After incubating overnight, the radii of zones of motility were measured.

### 2.6. Transmission Electron Microscopy

DRH212, *cbrR^−^* and *cbrR^+^* cells harvested from an overnight agar plate were washed in PBS and pelleted, then resuspended in 150 μL EM fixative (4% paraformaldehyde, 2% glutaraldehyde in 0.1 M cacodylate buffer) and incubated at 4 °C overnight. These samples were then negatively stained with 2% uranyl acetate and imaged with a JEM-1230 electron microscope (JEOL Inc, Peabody, MA, USA) run at 110 kV.

### 2.7. Autoagglutination Assay

In vitro assays to measure autoagglutination activity were performed as previously described [56]. *C. jejuni* cells from all three strains were harvested from overnight MH agar plates and washed in PBS before resuspending in PBS at an OD_600_ of 1.0. Two milliliters of each strain suspension was placed into glass culture tubes and incubated at room temperature in regular atmosphere and the OD_600_ of the top 1 mL measured at 2, 4, 6, 8, and 24 h timepoints.

### 2.8. Biofilm Assays and Scanning Electron Microscopy

Assays to quantify biofilm formation using crystal violet (CV) were performed as previously described [57]. Briefly, overnight agar plates of DRH212, *cbrR^−^* and *cbrR^+^* cells were harvested and suspended in MH at an OD_600_ of 0.1. Twenty-four-well plates were seeded with 1 mL bacterial suspension (for CV quantification). For CV visualization or SEM images of biofilm growth, 500 μL MH agar was dispensed into additional wells. After the agar hardened, 500 μL bacterial suspension was added, and 12 mm sterile glass coverslips were placed upright into the agar. Plates were then incubated in a microaerobic environment for 72 h. The wells for quantification were incubated with 1% CV in 20% ethanol, then aspirated and wells rinsed three times with distilled water, 80% DMSO was then added to solubilize the biofilm and was incubated overnight at room temp and absorption measured in a spectrophotometer at 570 nm. Coverslips for CV visualization were treated thusly: the bacterial suspension was aspirated and the coverslip was removed with sterile forceps; the coverslip was rinsed in successive beakers of distilled water twice and then immersed in 1% CV in 20% ethanol, after which coverslips were again rinsed in successive beakers of distilled water three times and allowed to dry. Coverslips for SEM had the bacterial suspension aspirated from the well, coverslip was removed with sterile forceps and rinsed in successive beakers of distilled water twice, and readied for SEM. Coverslips were submerged in EM fixative (same as that used in TEM experiments), then incubated at 4 °C until SEM imaging took place. Coverslips were then dehydrated, critical-point dried, sputter-coated using gold/palladium, and imaged with a Phillips XL-30 FEG microscope (ThermoFisher, Waltham, MA, USA).

### 2.9. Site-Directed Mutagenesis

Point mutations to the *cbrR* sequence in pCAF107 to identify critical amino acid residues were generated using the Q5 Site-Directed Mutagenesis Kit (NEB) according to manufacturer’s protocol. Primers CF114 and CF115 (to substitute an alanine residue for the predicted critical glutamic acid residue in the active site), and CF116 and CF117 (to inactivate the I-site by substituting the domain KGRD to AAAA) were designed using the NEBaseChanger (NEB) website to generate pCAF109 and pCAF110, respectively.

### 2.10. Differential Radial Capillary Action of Ligand Assay

Differential Radial Capillary Action of Ligand Assays (DRaCALA) were performed essentially as described [58]. Briefly, in a 96-well round-bottom plate, purified proteins (WspR- positive control for GTP and c-di-GMP binding, Alg44- positive control for c-di-GMP binding, CbrR, CbrR_KGRD(323-326)AAAA_, and CbrR_E334A_) were diluted in buffer (10 mM Tris, pH 8.0, 5 mM MgCl_2_, 100 mM NaCl). ^32^P-labeled GTP (1:100) and ^32^P-labeled c-di-GMP (1:2) were added to each protein and mixed via multi-channel pipet (to ensure similar mixing and timing. A pin tool was used to transfer solution to a flat nitrocellulose membrane by pressing firmly for 10–15 s. The membrane was allowed to dry completely and was imaged using a Fujifilm FLA-7000 scanner.

### 2.11. INT-407 Adherence/Invasion Assay

Measurement of *C. jejuni* adherence and invasion of INT-407 cells was performed as previously described [59]. To determine adherence to host cells, INT-407 cells (in RPMI with 10% FBS, 2 mM L-glutamine, 1% penicillin/streptomycin) were seeded in 24-well plates and incubated at 37 °C, 5% CO_2_ for 48 h until a monolayer of seeded cells was achieved. On the day of the experiment, cells were washed twice with pre-warmed Hank’s Balanced Salt Solution (HBSS) (MilliporeSigma, Burlington, MA, USA) and medium was replaced with maintenance medium (RPMI with 10% FBS, 2 mM L-glutamine, but no antibiotic/antimycotic). All three strains of *C. jejuni* used were grown overnight in biphasic cultures, pelleted, and resuspended in PBS at a bacterial concentration of 1 × 10^9^ bacteria/mL. CFU counts for each strain were performed by serially diluting the inoculum samples and plating out 100 μL of the 10^−5^, 10^−6^, and 10^−7^ dilutions. One-hundred microliters of the undiluted inoculum was used per well to infect the INT-407 cells and the plates were then centrifuged at 450× *g* for 15 min to remove differences in motility between strains. Cells were then incubated for three h. Wells were then washed three times with HBSS, then 1 mL 1% Triton X-100 in PBS and a micro magnetic stir bar was added to each well and cells were lysed on a stir plate for 10 min. Serial dilutions (1:10) of the lysate were made to the 10^−3^ and 100 μL were plated for CFU counting purposes. This represents adherent *C. jejuni*. To determine invasion of host cells, the same protocol to examine adherence was followed up to infection, at which point wells were washed twice with HBSS and maintenance medium with 50 μg/mL gentamicin was added and plates incubated for two h. Cells were then washed three times with HBSS and lysed in 1% Triton X-100 in PBS, serially diluted and plated just as adherence plates were. The percent of adherent and invasive cells were determined by comparison of the CFU counts to the inoculum samples.

### 2.12. Nucleotide Extractions for c-di-GMP Detection via LC-MS/MS

*C. jejuni* strains from plate, biphasic, and broth cultures were suspended or grown to a mid-log phase of OD_600_ of 0.6. *E. coli* BL21(DE3) cells transformed with pCAF107, pCAF109, pCAF110, and pCMW75 (positive control) and pCMW98 (negative control) were grown to an OD_600_ of 0.7, protein expression was induced with 1 mM IPTG, and grown for either 4 h at 37 °C or overnight at room temperature. Protein induction was confirmed on a 12% SDS-PAGE gel. Cells were pelleted and supernatant removed. Cells were washed in PBS, resuspended in 100 μL of extraction buffer (40% methanol, 40% acetonitrile, 0.1 N formic acid) and incubated at −20 °C for 30 min. Samples were then centrifuged at 16,000 RCF at 4 °C for 5 min and 90 μL of the solution was removed with care to avoid the pellet and placed in a clean 1.5 mL microcentrifuge tube. Tubes were placed in a speed vacuum overnight to dry the solvent and samples stored at −70 °C. Then, 10 μL of each sample was analyzed on a Quattro Premier XE (Waters Corp, Milford, MA, USA) mass spectrometer coupled with an Acquity Ultra Performance LC system. Using electrospray ionization, c-di-GMP was detected with MS parameters: capillary voltage, 3.5 kV; cone voltage, 50 V; collision energy, 34 V; source temperature, 110 °C; desolvation temperature, 350 °C; cone gas flow (nitrogen), 50 L/h; collision gas flow (nitrogen), 0.15 mL/min; and multiplier voltage, 650 V. Reverse phase chromatography separation used a Waters BEH C18 2.1 × 50 mm column with a 0.3 mL/min flow rate with a gradient of solvent A (10 mM tributylamine plus 15 mM acetic acid in 97:3 water:methanol) to solvent B (methanol): *t* = 0 min; A−99%:B−1%, *t* = 2.5 min; A−80%:B−20%, *t* = 7.0 min; A−35%:B−65%, *t* = 7.5 min; A−5%:B—95%, *t* = 9.01 min; A−99%:B−1%, *t* = 10 min. The c-di-GMP standard (Axxora) was dissolved in extraction buffer at 125, 62.5, 31.3, 15.6, and 7.8, 3.9, 1.9 nM concentrations to create a standard curve [60].

## 3. Results

### 3.1. C. jejuni CbrR Is Annotated as a DGC Similar to C. crescentus PleD

**Figure 1:**
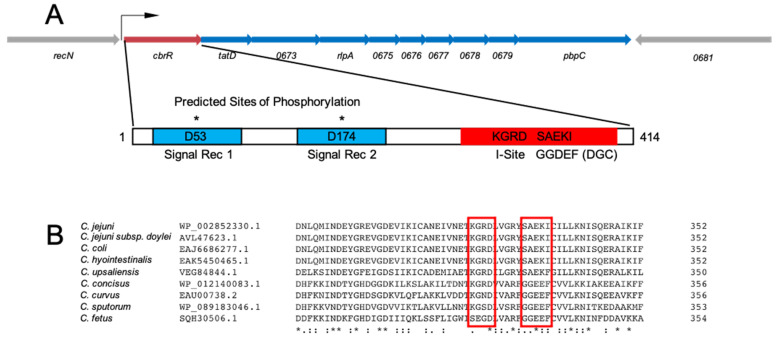
Gene and protein schematics for CbrR and the *cbrR* operon in *C. jejuni* 81–176. (**A**) The *cbrR* gene is the first of a predicted ten gene operon. For clarity, locus tags have been abbreviated to the last four digits. The bent arrow indicates the predicted transcription start site [68]. CbrR is 414 amino acid residues in length and is comprised of two N-terminal signal receiver domains (blue) with predicted sites of phosphorylation D53 and D174, and the GGDEF domain (red), which includes an autoinhibitory site (I-site). The GGDEF domain is indicative of diguanylate cyclase (DGC) activity. (**B**) A multiple sequence alignment shows that, though non-canonical, the amino acids comprising the I-site and active site are highly conserved within several clinically relevant Campylobacter species. Amino acid conservation is indicated by either an * (asterisk; fully conserved residues); a : (colon; amino acids with strongly similar properties; or a . (period; amino acids with weakly similar properties).

As annotated in the NCBI database, *C. jejuni* CbrR is a two-component response regulator that contains two N-terminal signal receiver domains and a C-terminal domain indicative of DGC activity (Figure 1). This includes an autoinhibitory site (I-site) and active site which contain highly variant sequences (KGRD and SAEKI, respectively) of the canonical RxxD and GG(D/E)EF, respectively (Figure 1). The divergent amino acids SAEKI are found in an alignment of catalytic sites found in proteins that retain DGC activity [61,62] and are highly conserved across the *Campylobacter* genus, including several species with clinical relevance (Figure 1). The I-site, located nine amino acid residues upstream of the active site, is predicted to belong to the class of domains which are known to negatively regulate production of c-di-GMP in DGCs while also aiding in protein–protein interactions [63]. CbrR also aligns with the closely related protein PleD in *Caulobacter crescentus*, which contains a similar domain arrangement to *C. jejuni* CbrR [18]. PleD is a response regulator with DGC activity that is involved in the transition from swarming to stalked cell morphology, which includes the ejection of the flagella and the beginning of cellular division [64,65], inducing biofilm formation and halting cell cycle progression [21]. Although an orphan response regulator that it is not associated with a cognate histidine kinase and lacking a DNA-binding domain, CbrR does have two predicted sites of aspartate phosphorylation on the N-terminal half of the protein, D53 and D174 (Figure 1), suggesting that phosphorylation is involved in activation/deactivation of the protein [18]. There is no crystal structure of CbrR; however, a Phyre2-predicted fold suggests the existence of a ligand-binding pocket and protein interface areas that bear striking resemblance to PleD in complex with c-di-GMP [66] (Supplementary Figure S2). This analysis also shows the degree of conservation of the C-terminal side of the protein based on Jensen–Shannon divergence [66,67] (Supplementary Figure S2).

### 3.2. CbrR Is Overexpressed in cbrR^+^ Complement

**Figure 2:**
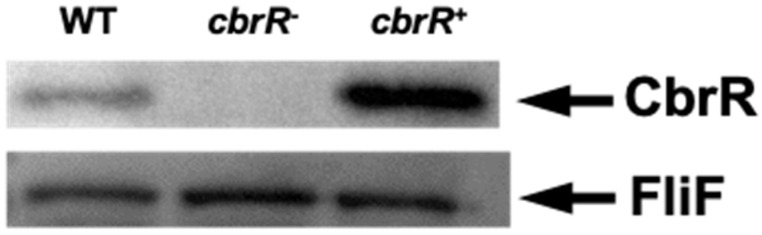
CbrR is absent in a *C. jejuni cbrR* mutant strain but overexpressed in the complemented strain. Western blots were performed on whole-cell lysates of WT, *cbrR* mutant (*cbrR^−^*), and complemented *cbrR^−^* mutant (*cbrR^+^*) cells. Western blots using antibodies to FliF, which is expressed equally in these strains, confirmed equivalent loading of the samples.

To study the effects of CbrR on *C. jejuni* phenotypes, we used streptomycin counterselection to construct an unmarked deletion of the *cbrR* gene [51], as well as a complemented mutant strain. The *cbrR^−^* mutant was complemented by inserting the gene with its native promoter via homologous recombination, along with a chloramphenicol resistance (*cat*) gene for selection, within the intergenic space between the rRNA subunit 16S and the gene for the tRNA-Ala molecule [69]. PCR showed the successful deletion and complementation of the *cbrR* gene in the mutant and complement strains (Supplementary Figure S3). To confirm the absence and presence of expression in *cbrR^−^* and *cbrR^+^*, respectively, we performed Western blots on all three strains. Expression of CbrR was missing from the mutant as expected; however, it was unexpectedly found in greater amounts in *cbrR^+^* when compared to the wild-type strain DRH212 (Figure 2). The reason for this unintentional increased expression of CbrR in the complemented strain *cbrR*^+^ is not clear but may be related to readthrough transcription from within the rRNA locus, as rRNA synthesis is differentially regulated by growth conditions [70].

### 3.3. Motility Is Enhanced in the Absence of CbrR

**Figure 3:**
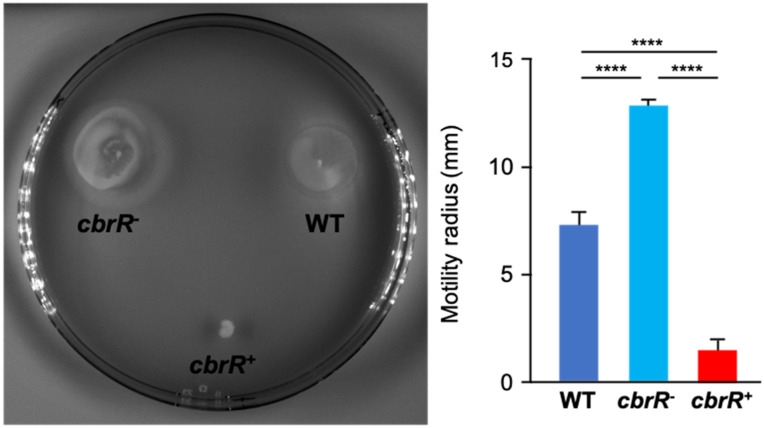
CbrR affects *C. jejuni* flagellar motility. Plate-grown *C. jejuni* WT, *cbrR^−^*, and *cbrR*^+^ cells were inoculated into 0.4% (*w*/*v*) agar and incubated overnight. Motility was measured by the radius of motile inoculum. The *cbrR^−^* mutant shows a hyper-motile phenotype relative to WT, while the complemented *cbrR^+^* strain shows a significant reduction in motility (**** *p* < 0.0001). A one-way ANOVA with Tukey’s multiple comparisons test was used for statistical analysis (GraphPad Prism 9, San Diego, CA, USA). This experiment was conducted three times in triplicate with similar results.

Cyclic-di-GMP and its regulatory role in motility was first found in 2004 [71] and regulation can occur by signaling a swarmer-to-stalked transition, but also by reversing flagellar rotation and switching cellular trajectory from “swimming” (straight, forward movement) to “tumbling” (random turning to change direction) [71,72]. As this dinucleotide is well known to interact with flagellar motor proteins, a suspected DGC would likely have a motility-associated phenotype. This, in addition to the fact that motility is acknowledged to be a critical virulence factor for *C. jejuni*, it was reasonably anticipated that a change in motility would be observed with dysregulated CbrR signaling. To ascertain this, we conducted motility assays in soft agar (0.4%), and the resulting zones of migration were measured after overnight incubations. Whereas DRH212 had a motility radius of 7.3 mm, the motilities of both *cbrR^−^* (12.8 mm) and *cbrR^+^* (1.5 mm) were significantly different from the wild type (Figure 3). The radii of motility showed that the elimination of CbrR showed an increase of 75% in the range of motility in the mutant strain, while cells that had increased expression of CbrR declined by 80% in the range of motility (Figure 3).

### 3.4. Higher Amounts of CbrR Lead to Loss of Flagellar Expression

**Figure 4:**
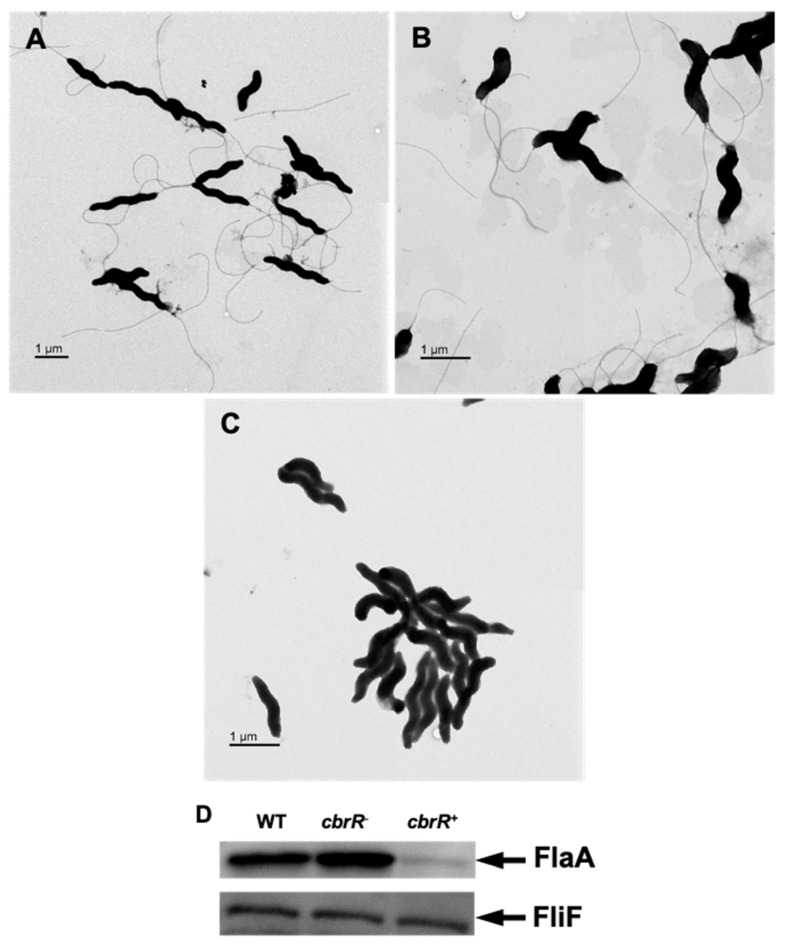
Flagellar expression is decreased with higher levels of CbrR. TEM images of WT cells (**A**) possess two polar flagella, as expected, as do *cbrR^−^* cells (**B**). However, overexpression of CbrR leads to the absence of flagella in *cbrR^+^* cells (**C**). This was confirmed via Western blot probing for FlaA in cell lysate from each strain, which showed greatly decreased expression of FlaA in *cbrR^+^* cells (**D**). Western blots using antibodies to FliF, which is expressed equally in these strains, confirmed equivalent loading of the samples.

**Figure 5:**
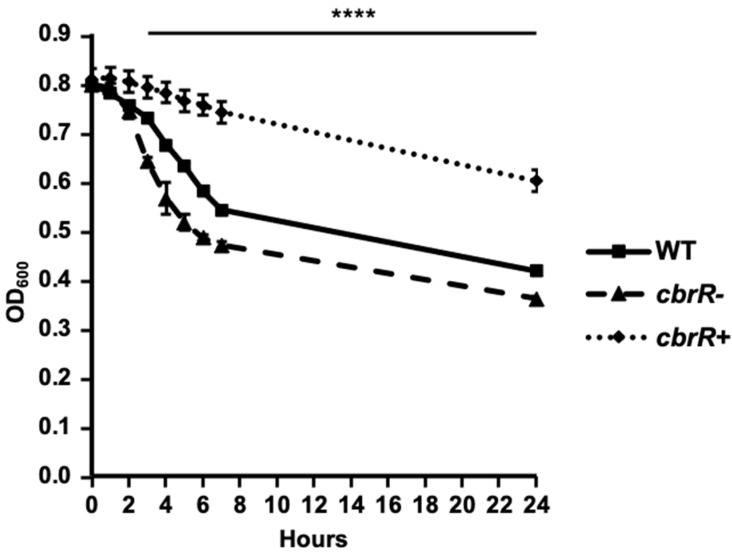
CbrR affects the extent of *C. jejuni* autoagglutination. AAG assays of WT, *cbrR^−^*, and *cbrR^+^* over 24 h. The AAG assay measures the tendency of cells suspended in PBS to aggregate via the flagella over time by measuring the OD_600_ in the top 1 mL of the 2 mL volume suspension of cells. The *cbrR^−^* mutant shows a faster tendency to autoagglutinate when compared to WT, while the complemented *cbrR^+^* shows delayed autoagglutination. All three groups were statistically different from 3 h onward (**** *p* < 0.0001). A one-way ANOVA with Tukey’s multiple comparisons test was used to determine statistical significance (GraphPad Prism 9, San Diego, CA, USA). This experiment was performed in triplicate and conducted three times.

Due to the effect of varied CbrR levels on motility, it was important to examine the cellular morphology of the three strains to determine if flagellar biosynthesis was affected. To achieve this, we acquired transmission electron microscopy (TEM) images of all three strains which had been cultured on agar plates overnight. While both the DRH212 and *cbrR^−^* strains retained the typical bipolar flagellar arrangement (Figure 4A,B), *cbrR^+^* cells showed a lack of flagella (Figure 4C). To confirm this finding, Western blots for flagellin expression in cell lysates of the three strains were probed with anti-FlaA antiserum. Results indicated a slight increase in FlaA expression in *cbrR^−^* lysate when compared to DRH212, whereas flagellin expression in *cbrR^+^* was demonstrably lower than both WT and *cbrR^−^* lysates (Figure 4D), consistent with the electron microscopy findings. As *cbrR^+^* cells showed fewer flagella in TEM images and reduced flagellin expression in Western blots, we examined the aggregation of bacterial cells, or autoagglutination (AAG), which in *C. jejuni* is achieved by the clumping of cells via their heavily glycosylated flagella [39]. This is determined by measuring the rate at which the bacteria settle in solution [56]. Results indicated that *cbrR^−^* cells have an increased rate of AAG compared to WT, whereas the *cbrR^+^* strain showed a significant defect at all timepoints (Figure 5).

### 3.5. CbrR Affects the Ability of C. jejuni to Attach to and Invade Human INT407 Cells

**Figure 6:**
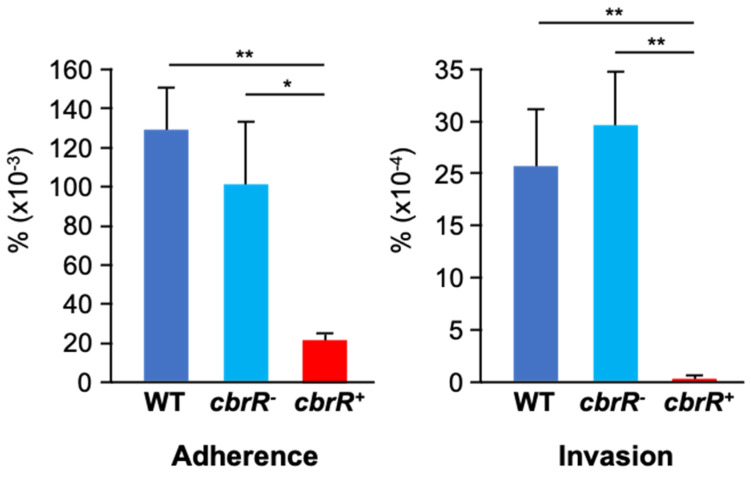
Loss of CbrR expression leads to a defect in host cell adherence and invasion. Assessment of WT, *cbr^−^R*, and *cbrR^+^* strains to attach and invade host cells was determined by infecting human epithelial INT407 cells with each of the strains. WT and *cbrR^−^* cells attached and invaded cells at similar rates, whereas *cbrR^+^* cells showed deficiencies in both adherence and invasion compared to both WT and *cbrR^−^* (* *p* < 0.05; ** *p* < 0.01). Statistical significance was determined by a one-way ANOVA with Tukey’s multiple comparisons test (GraphPad Prism 9, San Diego, CA, USA). Experiments were performed three times in triplicate.

*C. jejuni* adherence to host cells has been shown to be mediated by the flagellar tip [35,36] and is necessary for invasion of host cells. Motility is also a required phenotype for efficient colonization [30]. As the overexpression of CbrR is associated with loss of flagellar expression and motility, we next determined whether it affected *C. jejuni* attaching to and invading host cells. DRH212 and *cbrR^−^* cells had similar rates of attachment at 0.13% and 0.1%, respectively; however, *cbrR^+^* cells showed a deficiency in adherence at 0.02% (Figure 6). This same trend was seen in cellular invasion, with DRH212 and *cbrR^−^* cells invading at 0.0024% and 0.0029% (respectively), while *cbrR^+^* cells were found within only 0.0001% of INT407 cells. These assays indicated that WT and *cbrR^−^* cells both attached and invaded cells at a similar magnitude; however, *cbrR^+^* cells were deficient in adherence and unable to invade human INT407 cells efficiently (Figure 6).

### 3.6. CbrR Negatively Regulates Biofilm Formation

**Figure 7:**
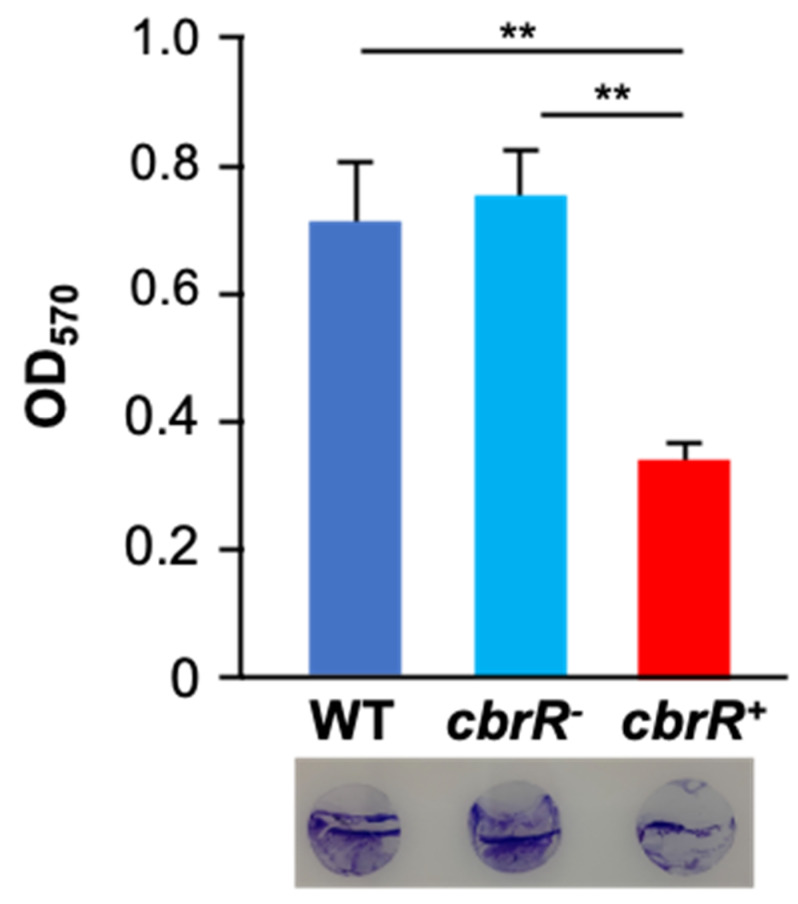
Overexpression of CbrR leads to a decrease in biofilm formation. Quantitative and qualitative assessment of biofilm formation by WT, *cbrR^−^*, and *cbrR^+^* was performed using crystal violet staining of 72 h *C. jejuni* biofilms grown in 24-well microtiter plates. The graph shows biofilm formed (measured by OD_570_), while the circles at the bottom are crystal violet-stained coverslips representative of those subjected to SEM. Wild type and *cbrR^−^* cells retain the ability to effectively form biofilm; however, overexpression of CbrR leads to a biofilm formation deficiency in *cbrR^+^* (** *p* < 0.01). One-way ANOVA with Tukey’s multiple comparisons test was performed for statistical analysis (GraphPad Prism 9, San Diego, CA, USA). Experiments were performed with triplicate samples and repeated three times.

**Figure 8:**
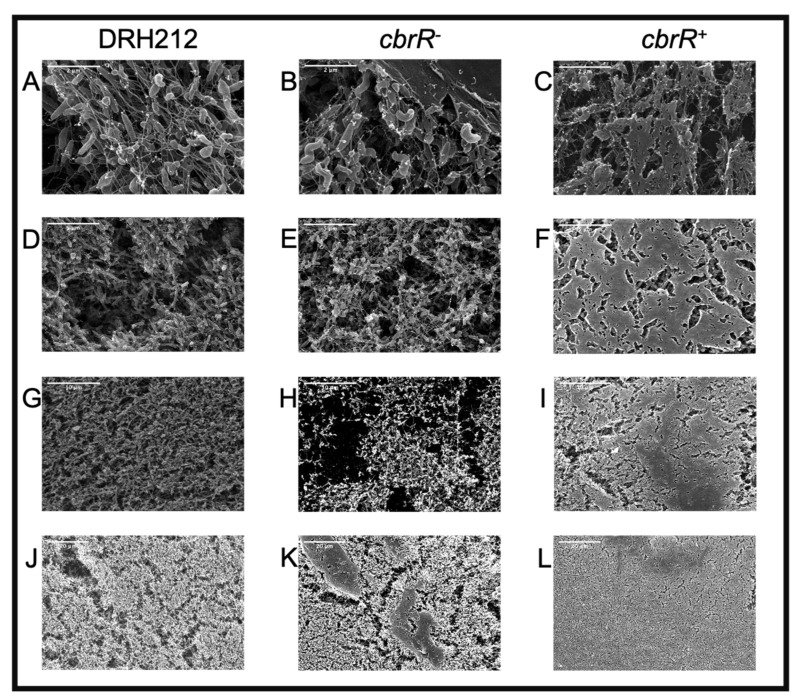
Robust biofilm formation is seen in wild type and *cbrR^−^* but is absent in *cbrR^+^*. SEM images at different magnifications (25,000×: **A**–**C**; 10,000×: **D**–**F**; 5000×: **G**–**I**; 2000×: **J**–**L**) of coverslips propped in *C. jejuni* inoculated biphasic cultures for 72 h. Images show abundant biofilm formation in DRH212 (**A**,**D**,**G**,**J**) and *cbrR^−^* (**B**,**E**,**H**,**K**) cells, whereas reduced accumulation of biomass is seen in *cbrR^+^*(**C**,**F**,**I**,**L**).

Because motility is required for robust biofilm formation in *C. jejuni* [43], we next determined the effect of CbrR on biofilm formation. We performed static biofilm assays on the three strains using crystal violet (CV) staining to quantify biofilm production, and to visualize biofilm on glass coverslips (Figure 7). Both DRH212 and *cbrR^−^* strains formed comparable biofilms with substantial accumulation of cells with optical density at 570 nm (OD_570_) of 0.87 and 0.92, respectively, whereas *cbrR^+^* showed a significant reduction in biofilm formation with OD_570_ of 0.42 (Figure 7). SEM images of biofilm formed at the air-liquid surface interface on coverslips showed robust biofilm formation in DRH212 cells, with dense biomass covering almost the entirety of the surface (Figure 8J) and what can be described as the “fisherman’s net” usually seen in *C. jejuni* biofilm (Figure 8A). This same rich matrix was seen in *cbrR^−^* biofilms, including significant numbers of cells (Figure 8K). Profuse netting was seen in the *cbrR_−_* samples (Figure 8B), similar to that of DRH212 cells (Figure 8A). In contrast, *cbrR^+^* biofilms were seen with far fewer cells (Figure 8C,F,I,L), with swaths of bare agar seen (Figure 8F,I,L), though some net-like matrix was produced with few cells present within (Figure 8C).

### 3.7. Purified CbrR Is Able to Bind Both GTP and c-di-GMP

**Figure 9:**
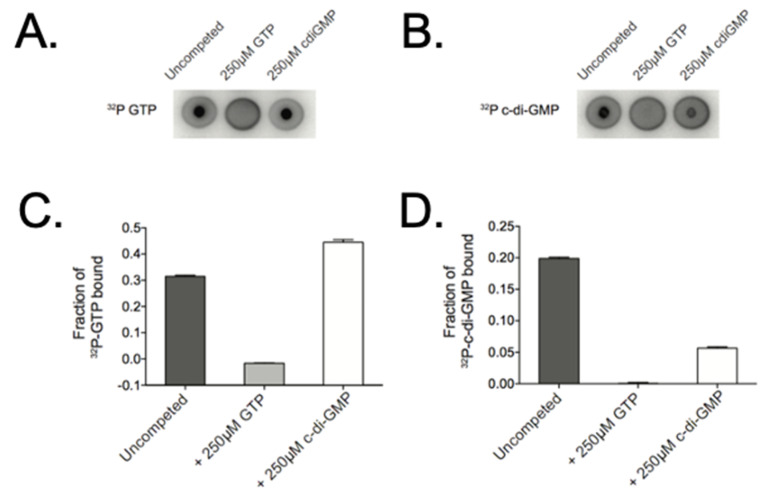
CbrR binds both GTP and c-di-GMP. To ascertain the ability of CbrR to bind nucleotides, purified CbrR was mixed with radio-labeled GTP and c-di-GMP and spotted on a nitrocellulose membrane. Imaging of proteins that bind radio-labeled nucleotide show a dark, intense spot in the middle of the membrane, while nucleotide that does not bind protein diffuses across the membrane to appear as a dark circle around the membrane where the solution was spotted. DRaCALA assays show that CbrR binds GTP (**A**) and c-di-GMP (**B**) in a similar manner. Competition binding shows a slightly higher affinity of CbrR binding GTP over c-di-GMP (**C**,**D**).

The formation of c-di-GMP by a DGC is a two-step process, beginning with binding of the substrate GTP, and ending with the product, c-di-GMP. Due to the fact that the amino acid sequence of the CbrR active and autoinhibitory sites were divergent, it was unknown whether it was capable of binding these nucleotides. We therefore determined the binding of GTP and c-di-GMP by CbrR, using Differential Radial Capillary Action of Ligand Assays (DRaCALA). Using purified CbrR and radiolabeled GTP and c-di-GMP, this assay indicated efficient binding of both GTP and c-di-GMP, as demonstrated by a dark circle in the middle of the spot indicating a nucleotide-protein complex bound to the membrane and immobilizing the labeled nucleotide (Figure 9A,B). In competition experiments, GTP seemed to outcompete c-di-GMP for CbrR binding, as indicated by the fact that the addition of unlabeled GTP prevented the binding of the ^32^P-GTP to the protein and diffused across the membrane while the addition of unlabeled c-di-GMP did not (Figure 9A). This was also quantified by densitometry (Figure 9C). Unlabeled GTP outcompeted ^32^P-c-di-GMP for binding sites, as indicated by the loss of the visible protein complex in the middle of the membrane, whereas unlabeled c-di-GMP did not completely prevent binding of ^32^P-c-di-GMP, resulting in both a dark spot in the middle and a ring around the perimeter (Figure 9B) with corresponding densitometry (Figure 9D).

### 3.8. Mutation of the Autoinhibitory Site Leads to Loss of c-di-GMP Binding

**Figure 10:**
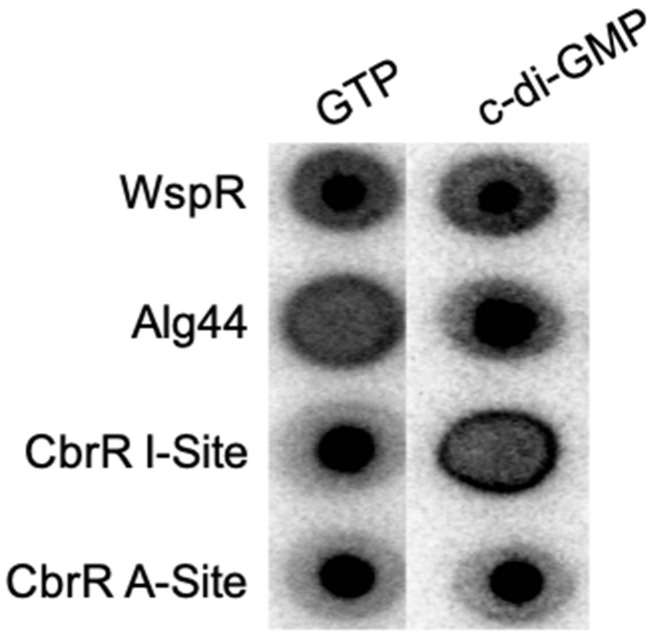
Mutation in the autoinhibitory site of CbrR leads to loss of c-di-GMP binding. Point mutants of *cbrR* to inactivate the I-site and active site were generated and their ability to bind GTP and c-di-GMP were established in DRaCALA assays. Results show that the CbrR active site mutant binds both GTP and c-di-GMP; however, the I-site mutant lost the capacity to bind c-di-GMP. The diguanylate cyclase *Pseudomonas fluorescens* WspR is shown as a positive control able to bind both small molecules while *Pseudomonas aeruginosa* Alg44 is a negative control for GTP binding but a positive control for c-di-GMP binding.

As the I-site in DGCs serves to negatively regulate the synthesis of c-di-GMP when it binds the product, and the active site is responsible for the enzymatic activity, we determined whether inactivation of either site would inhibit small molecule binding. We performed site directed mutagenesis (SDM) on both domains in pCAF107 to substitute specific amino acids residues to alanine (E334 in the predicted active site and K323-D326 in the I-site) for expression and purification. DRaCALA assays on these mutant CbrR proteins showed that substituting the predicted essential amino acid in the active site did not affect binding of either GTP or c-di-GMP seen in the dark spots in the middle of the membrane for both molecules (Figure 10); however, the substitution of the I-site in the protein led to the loss of c-di-GMP binding, as indicated by the loss of the intense spot in the middle of the c-di-GMP sample (Figure 10). This indicates that the I-site of CbrR binds c-di-GMP.

### 3.9. Low Levels of c-di-GMP Were Detected in C. jejuni and in E. coli

**Figure 11:**
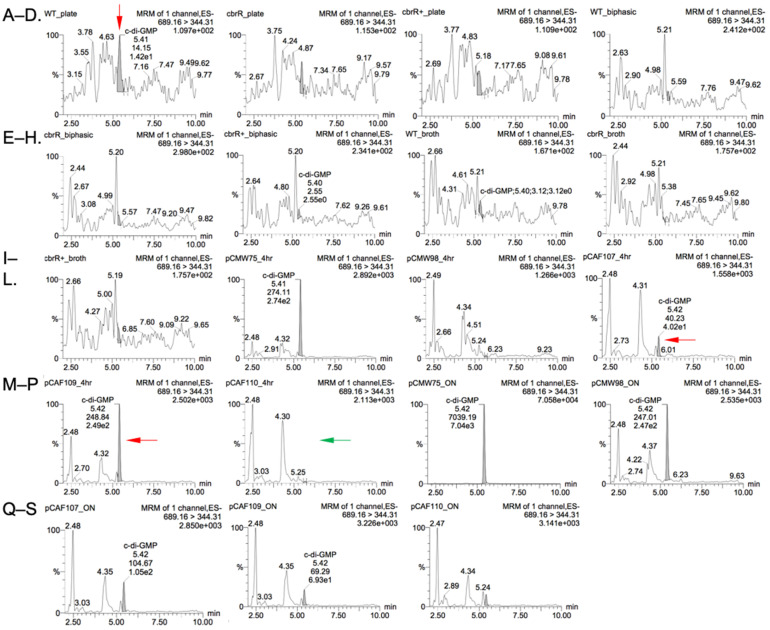
LC-MS/MS shows low level amounts of c-di-GMP produced by *C. jejuni* CbrR. Chromatograms generated by LC-MS/MS show peak intensity of c-di-GMP for samples of *C. jejuni* cells: (**A**) WT Plate, (**B**) *cbrR^−^* Plate, (**C**) *cbrR^+^* Plate, (**D**) WT Biphasic, (**E**) *cbrR^−^* Biphasic, (**F**) *cbrR^+^* Biphasic, (**G**) WT Broth, (**H**) *cbrR^−^* Broth, (**I**) *cbrR^+^* Broth; and *E. coli* cells: (**J**) pCMW75 4 Hr, (**K**) pCMW98 4 Hr, (**L**) pCAF107 4 Hr, (**M**) pCAF109 4 Hr, (**N**) pCAF110 4 Hr, (**O**) pCMW75 O/N, (**P**) pCMW98 O/N, (**Q**) pCAF107 O/N, (**R**) pCAF109 O/N, (**S**) pCAF110 O/N. WT *C. jejuni* and recombinant *E. coli* (WT CbrR or CbrR_E334A_) samples showed low level c-di-GMP production.

As a putative DGC with variant sequences in both the active site and I-site, we used nucleotide extraction and liquid chromatography with tandem mass spectrometry (LC-MS/MS) to attempt to detect and measure production of c-di-GMP by *C. jejuni* CbrR. Nucleotide extractions were performed on all three strains of *C. jejuni* cells that were grown on agar plates, in biphasic cultures, and in broth, as the conditions under which CbrR is activated is unknown. Additionally, the expression vectors used for the DRaCALA assays and controls (the known *Vibrio harveyi* DGC, QrgB in pCMW75 and an inactive mutant of QrgB in pCMW98) were expressed in BL21(DE3) *E. coli* cells and nucleotide extractions performed. LC-MS/MS results indicated slight detection in the WT, plate-grown sample that was well below the lowest concentration of purified c-di-GMP used to generate a standard curve, but no detection in any of the other *C. jejuni* samples (Table 1 and Figure 11A–I). *E. coli* samples of the control DGC QrgB (pCMW75) indicate a robust, positive result of 816 nM c-di-GMP detected (Table 1 and Figure 11O). Expressed versions of CbrR (pCAF107 and pCAF109) showed detectable amounts of c-di-GMP (Table 1 and Figure 11L,M, respectively, red arrows), whereas the I-site mutant expressed from pCAF110 did not produce c-di-GMP (Table 1 and Figure 11N, green arrow).

## 4. Discussion

Though *Campylobacter* was first described in the 1800s, the exact mechanism by which these bacteria cause hundreds of millions of cases of diarrhea each year is largely unknown, though several virulence factors have been uncovered [2,7,38,73,74,75]. Motility and chemotaxis are considered crucial to *C. jejuni* pathogenesis [30,75,76,77,78,79]. Therefore, fully understanding the regulation of these two factors must be carefully explored and unraveled in the hopes of finding a prophylactic treatment to prevent campylobacteriosis and *C. jejuni*-associated sequelae. Another putative virulence factor is CbrR, which mediates bile resistance and is involved in chick colonization [18]. Here we show that CbrR inhibits motility in *C. jejuni*. RNA-Seq experiments have previously shown that *cbrR* was downregulated during chick colonization [26,27], supporting the consensus that motility is required for colonization and infection. As bile is a known *C. jejuni* chemorepellent [80,81,82], and yeast two-hybrid experiments showed that *C. jejuni* CbrR interacts with CheA (a critical chemotaxis protein that is central to the chemotactic response) [29], CbrR has the potential to modulate both motility and chemotaxis.

Many of these characteristics have been shown to be regulated in other bacteria, at least in part, by c-di-GMP signaling. We therefore explored a potential role for CbrR, and c-di-GMP production, in regulating *C. jejuni* motility/chemotaxis. Bioinformatic analysis of the amino acid sequence indicates that *C. jejuni* CbrR is a possible DGC, though the active site and I-site sequences are highly variant from the consensus (Figure 1). An unmarked deletion of the gene and subsequent complementation of the mutation indicated a dysregulation of motility associated with either deletion or overexpression of CbrR (Figure 3). Complementation of the *cbrR* mutation in the rRNA locus led to the unexpected overexpression of CbrR, and this was seen consistently in numerous experiments. While *cbrR* was expressed from its own promoter, we suspect that CbrR overexpression resulted from readthrough transcription from rRNA locus promoters that were highly active under the conditions used here. This overexpression of CbrR revealed its negative regulatory effect on FlaA expression. The downregulation of FlaA expression upon overexpression of CbrR, and concomitant reduction in the presence of flagella (Figure 4), presumably explains the significant reduction in motility in the complemented mutant *cbrR^+^*.

Biofilm formation is a critical survival mechanism for this microaerophilic organism, leading to increased horizontal gene transfer and, therefore, higher antibiotic resistance [42]. *C. jejuni* biofilms are important on poultry farms where it colonizes the intestinal tract of the chicken [45], while also allowing it to persist in environments to which it is ill-suited until such a time when conditions are sufficient to disperse from the biofilm and go on to colonize or infect a new host [42,43,44,48,50]. It is also known that flagellar defects can lead to a biofilm-deficient phenotype [48]. As such, it is not surprising that the flagellar-deficient *cbrR^+^* was unable to form the robust biofilm seen in both WT and *cbrR^−^* strains (Figure 7 and Figure 8). However, it is not conclusive whether this is a direct result of fewer flagella or inhibited motility. The fact that *cbrR^+^* showed a deficiency in AAG kinetics (Figure 5), which is mediated by flagellar contact and is one of the initial steps in biofilm formation [39,50], suggests that the inability to form biofilm is due to the reduced number of flagella seen in the complemented mutant (Figure 4). This loss in both flagellar expression and motility associated with *cbrR^+^* is likely also responsible for the inability of the strain to attach to and invade INT407 cells to the same degree as WT and *cbrR^−^* (Figure 6), supporting findings that host cell adherence is mediated by the flagellar tip [35,36] and indicating a substantial role for CbrR in pathogenicity in addition to its role in bile resistance.

Having established a role for CbrR in *C. jejuni* motility and pathogenesis, we addressed the overarching question of whether CbrR is part of a c-di-GMP signaling system in *C. jejuni*. While annotated as a DGC, the I-site (KGRD) and active site (SAEKI) of CbrR are rather divergent from the consensus sequences of DGCs. In many such instances, this indicates an evolutionarily ‘decommissioned’ DGC that is no longer enzymatically active but rather functions as a c-di-GMP receptor [71]. Whether CbrR is a bona fide DGC or a c-di-GMP receptor, it stands to reason that if CbrR were functionally inactive it would not be so highly conserved across the *Campylobacter* genus [83] (Figure 1B).

To distinguish between these possibilities and determine whether a c-di-GMP signaling system exists in *C. jejuni*, we first examined potential c-di-GMP binding and production by CbrR. DRaCALA experiments (Figure 9) clearly showed that wild-type CbrR bound both the DGC substrate GTP and its product c-di-GMP. Therefore, although both the active site and I-site are comprised of variant amino acid sequences relative to consensus, CbrR retained GTP and c-di-GMP binding ability (Figure 10), indicating that it may be able to participate in a c-di-GMP signaling pathway. Subsequent DRaCALA experiments (Figure 10) using site-directed mutants of CbrR (either E334A or KGRD(323-326)AAAA) showed that c-di-GMP is bound at the I-site, as expected if CbrR is either a DGC or a c-di-GMP receptor protein.

LC-MS/MS experiments showed low-level production of c-di-GMP in wild-type *C. jejuni* grown on plates, but not in broth (Figure 11A, Table 1). The reason for such low-level production is not clear; however, it is possible that CbrR exerts its effects on *C. jejuni* motility in a highly localized manner such that large amounts of c-di-GMP are not produced cell-wide. Furthermore, it is possible that c-di-GMP production by CbrR is regulated by environmental conditions, with higher production under conditions different than those tested here. Production of c-di-GMP by CbrR is further substantiated in *E. coli* expressing wild-type CbrR (Figure 11L, Table 1), by CbrR containing an E334A mutation (Figure 11M, Table 1), but not by CbrR containing a complete KGRD(323-326)AAAA substitution of the I-site (Figure 11N, Table 1). The loss of c-di-GMP production by the complete I-site replacement may have resulted from disruption of the adjacent active site by the four-amino acid substitution. Taken together, however, these data suggest that CbrR is capable of synthesizing c-di-GMP in *E. coli*, albeit at low levels. The reasons for the apparent low level of c-di-GMP production by CbrR in *E. coli* are unclear; however, several possibilities exist. Enzymatic activities of DGCs are often dependent upon activation of the protein, availability of substrate and cofactors, and can even be dependent upon growth cycle stage [84]. This can be extended to include necessary protein–protein interactions to effectively stimulate c-di-GMP synthesis by DGCs. For example, c-di-GMP production by the DGC SadC in *P. aeruginosa* can be activated by interaction with the flagellar stator MotC [85]. In this system, it is thought that c-di-GMP signaling leads to the dislocation of MotCD from the flagellar motor, which then interacts with SadC to further increase c-di-GMP when motility is not warranted. It is therefore possible that similar CbrR^−^ activating factors from *C. jejuni* (not present in *E. coli*) could be required to provide higher level c-di-GMP production by CbrR expressed in *E. coli*. It is also possible that CbrR is somehow activating the production of c-di-GMP by resident *E. coli* DGCs, although it is not clear how this would be achieved by wild-type CbrR but not CbrR(KGRD(323-326)AAAA).

In the absence of high-level c-di-GMP production, it is also possible that CbrR serves primarily as a c-di-GMP receptor, as has been seen in other organisms. Enzymatically inactive DGCs and PDEs found in *E. coli* still regulate biofilm production and motility through protein–protein and small regulatory RNA (sRNA) interactions which can be regulated by nucleotide binding to the degenerative active sites [86]. It has also been proposed that DGCs with degenerative active sites that contain I-sites become specific effectors with regulatory roles in the cells, such as with PopA in *C. crescentus*, which is activated upon binding c-di-GMP to localize a cell cycle regulatory protein for degradation [87]. A similar role for CdgG in *Vibrio cholerae* is seen in regards to biofilm formation and motility [87]. The same is seen for degenerative PDEs: the binding of c-di-GMP by FimX in *P. aeruginosa* serves to regulate twitching motility [88]. Taken together, classes of degenerative DGCs and PDEs working as c-di-GMP effectors to induce phenotypic changes in a cell in response to the presence of this signaling molecule leads to the conclusion that the conservation of these genes that produce degenerative enzymatic domains within the bacterial chromosome is necessary for optimal survival.

The binding of c-di-GMP by the highly conserved CbrR strongly suggests the presence of a c-di-GMP-responsive system in *C. jejuni*; however, such a system remains ill-defined. Other than CbrR, there are no annotated PDEs or c-di-GMP receptors, although such proteins could be cryptic. However, it is also possible that *C. jejuni* depends on surrounding bacteria to produce c-di-GMP that it senses and responds to much in the way of chemotactic signaling or quorum sensing. There is evidence of bacterial response to exogenous c-di-GMP in a reduction in cell aggregation and biofilm formation in *Staphylococcus aureus* [89,90]. A similar effect of treatment with c-di-GMP on *S. aureus* was seen when injections of the signaling molecule into the mammary gland prevented colonization of the mammary tissue in a dose-dependent manner [90,91]. In this sense, it is possible that bacteria could communicate environmental cues via a paracrine signaling system.

In this work, we show that the annotated DGC CbrR is a negative regulator of motility in *C. jejuni*. This reduction in motility is associated with lower expression of the major flagellin FlaA, altered flagellar number, and changes in AAG. Lower flagellar expression also results in a reduced ability to form robust biofilm, which may be attributable to the inability of these cells to aggregate via AAG to form microcolonies. Further, overexpression of CbrR led to the reduction in both virulence and pathogenicity, due to a decreased ability to attach to and invade human INT407 cells. While production of c-di-GMP by *C. jejuni* CbrR was detected at low levels, the significance of which is unclear, this protein is still able to bind both GTP and c-di-GMP, the substrate and product of DGC activity. These studies have therefore provided evidence of a novel c-di-GMP regulatory system in the pathogen *C. jejuni*. As all of the phenotypes thus far discovered to be regulated by CbrR are associated with survival and virulence, this protein could be a prime target as a possible prophylactic therapy to prevent the colonization and/or infection of host organisms.

## Figures and Tables

**Table 1:** Detection of c-di-GMP in nucleotide extraction samples.

Sample	c-di-GMP (nM) in Cellular Extracts
WT Plate	0.4
*cbrR^−^* Plate	0
*cbrR^+^* Plate	0
WT Biphasic	0
*cbrR^−^* Biphasic	0
*cbrR^+^* Biphasic	0
WT Broth	0
*cbrR^−^* Broth	0
*cbrR^+^* Broth	0
QrgB (pCMW75) 4 h expression	30.6
Inactive QrgB (pCMW98) 4 h expression	0
WT CbrR (pCAF107) 4 h expression	3.4
Active site mutant CbrR (pCAF109) 4 h expression	27.6
I-site mutant CbrR (pCAF110) 4 h expression	0
QrgB (pCMW75) O/N expression	816.0
Inactive QrgB (pCMW98) O/N expression	27.4
WT CbrR (pCAF107) O/N expression	10.9
Active site mutant CbrR (pCAF109) O/N expression	6.8
I-site mutant CbrR (pCAF110) O/N expression	4.0
c-di-GMP Standard (1.953 nM)	3.3
c-di-GMP Standard (3.906 nM)	4.2
c-di-GMP Standard (7.813 nM)	7.1
c-di-GMP Standard (15.625 nM)	15.6
c-di-GMP Standard (31.250 nM)	28.5
c-di-GMP Standard (62.500 nM)	63.8
c-di-GMP Standard (125.000 nM)	125.4
Blank	0

## Data Availability

Not applicable.

## Supplementary Materials

The following are available online at https://www.mdpi.com/article/10.3390/microorganisms10010086/s1, Figure S1: Purification of WT and mutant CbrR, Figure S2: Predicted protein structure of *C. jejuni* CbrR using the known crystal structure for ortholog *Caulobacter crescentus* PleD, Figure S3: Replacement of *cbrR* confirmed by PCR, Table S1: Bacterial strains and plasmids used for this study, Table S2: PCR primers used for this study.
