# Supplementary material for: The Campylobacter jejuni Response Regulator and Cyclic-Di-GMP Binding CbrR Is a Novel Regulator of Flagellar Motility
Source: Microorganisms. 2021 Dec 31;10(1):86. doi: 10.3390/microorganisms10010086 (PMC8779298; doi:10.3390/microorganisms10010086)
Supplement: Supplementary file 1 [file microorganisms-10-00086-s001.zip › microorganisms-1463694-supplementary.pdf]

Figure S1

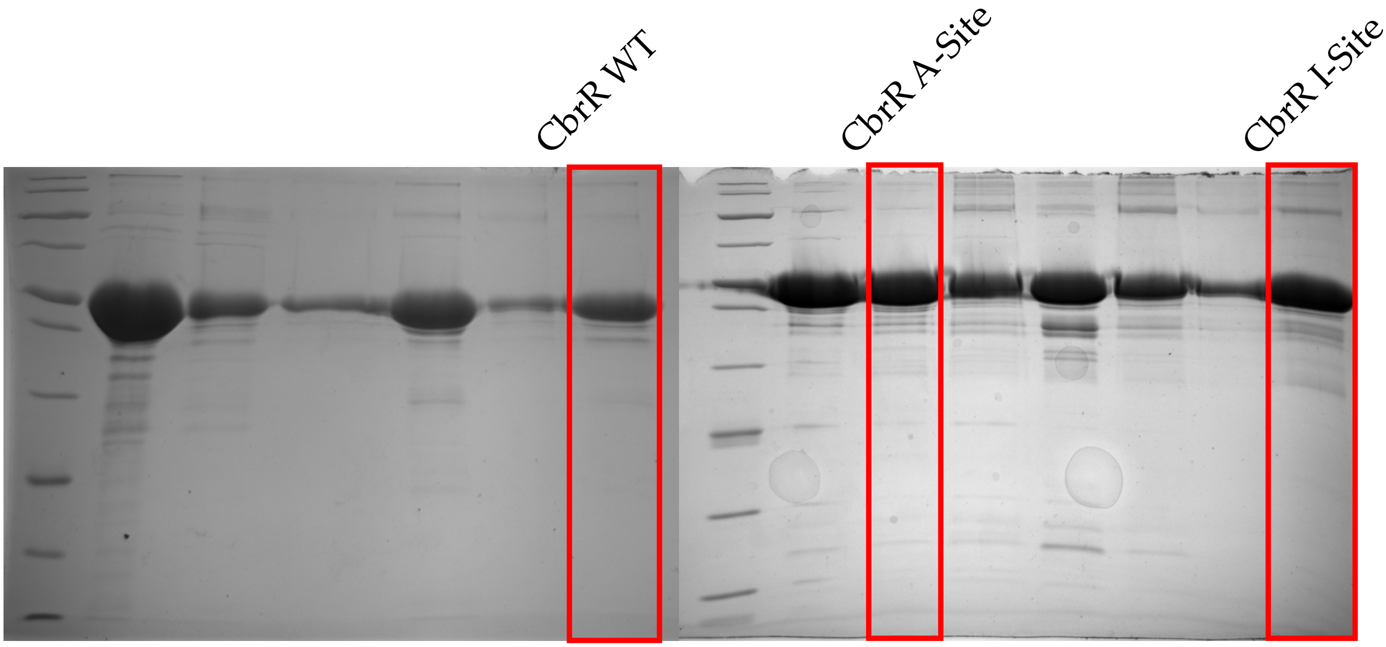

Figure S1. Purification of WT and mutant CbrR. To express and purify a His-tagged recombinant protein, cbrR was amplified from pCAF101 with primers CF112 and CF113 and subcloned into pET-20b(+), creating pCAF107. Site-directed mutagenesis was then used to introduce point mutations using primers CF114, CF115 (to substitute an alanine residue for the glutamic acid residue tin the active site) and CF116, CF117 (to substitute an AAAA sequence for the KGRD sequence of the autoinhibitory site) resulting in pCAF109 and pCAF110, respectively.

Figure S2

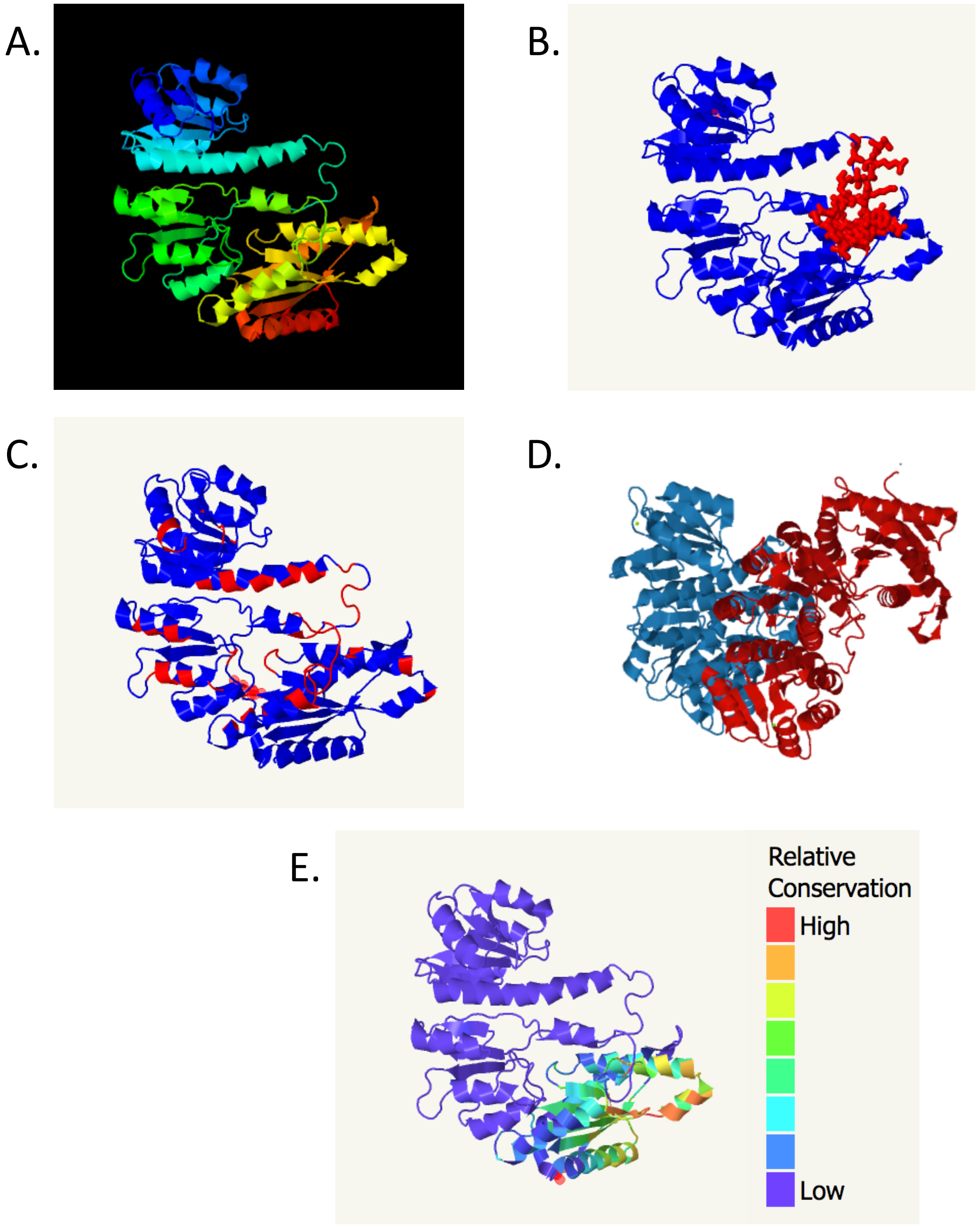

Figure S2. Predicted protein structure of *C. jejuni* CbrR using the known crystal structure for ortholog *Caulobacter crescentus* PleD
A) CbrR is predicted to have 12 alpha-helices and 12 beta-strands, B) with a large predicted pocket (red) and C) sites of protein-protein interaction (red) in an orientation that would suggest homodimerization comparable to that seen in D) PleD and E) a high degree of amino acid conservation on the C-terminus.

Figure S3

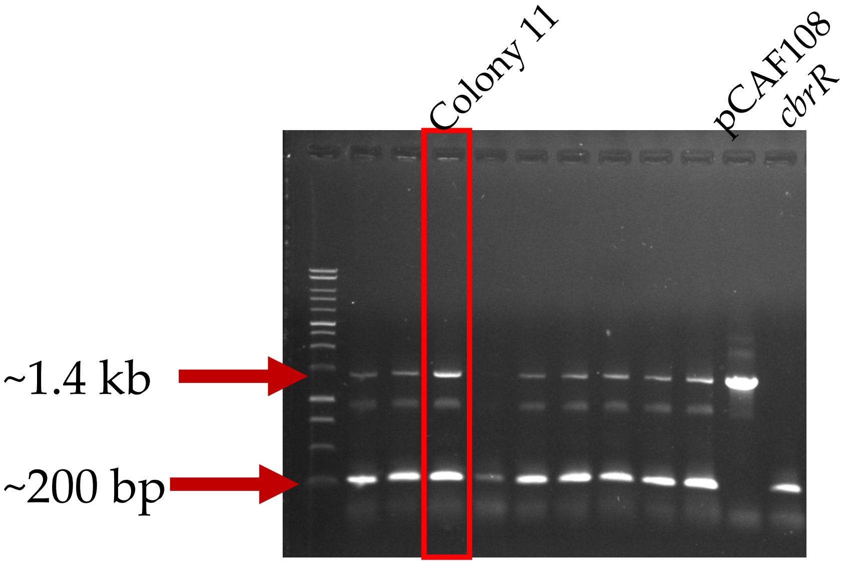

Figure S3. Replacement of *cbrR* confirmed by PCR. Complementation of the *cbrR* gene within the *cbrR* mutant in colony 11 confirmed by PCR using primers CF110 and CF111 which would indicate the successful reintroduction of *cbrR* (1.4 kb band) or not (200 bp band); pCAF108 was used to indicate where a positive band would amplify, *cbrR* DNA was used to show the deleted region which would also amplify.

Table S1. Bacterial strains and plasmids used for this study

Strain or Plasmid	Description	Resistance	Source or Reference
**Strain**			
Campylobacter jejuni			
**81-176**	Source of chromosomal DNA		[1]
**DRH212**	Wild type	Str	[2]
cbrR^{-}	Deletion mutant of DRH212	Str	This study
cbrR^{+}	Complemented *cbrR* strain	Str, Cm	This study
Escherichia coli			
**One Shot Top 10**	Cloning strain		Thermo
**DH5α**	Cloning strain		Thermo
**C2987**	Cloning strain		Invitrogen
**BL21(DE3)**	Protein expression strain		Promega
**Plasmids**			
**pCRII-TOPO**	Cloning vector	Amp, Km	Invitrogen
**pET-20b(+)**	Cloning vector	Amp	Invitrogen
**pCAF101**	*cbrR* with flanking regions in pCRII-TOPO	Amp, Km	This study
**pCAF102**	pCAF101 after removal of *cbrR* coding region, but retaining flanking regions	Amp, Km	This study
**pCAF103**	$rpsL_{HP}$-*cat* cassette in pCRII-TOPO	Amp, Km, Cm	This study
**pCAF104**	pCAF102 with $rpsL_{HP}$-*cat* cassette from pCAF103 placed within the *cbrR* flanking regions	Amp, Km, Cm	This study
**pCAF107**	pET-20b(+) with coding sequence for CbrR to create CbrR-His_6	Amp	This study
**pRRC**	Shuttle vector for *C. jejuni* gene delivery	Amp, Cm	[3]
**pCAF108**	pRRC with *cbrR* with native promoter	Amp, Cm	This study
**pCAF109**	pCAF107 with point mutation E334A	Amp	This study
**pCAF110**	pCAF107 with point mutation substituting alanine residues for entire I-site (K323-D326)	Amp	This study

**pCMW75**	Expression vector for *V. harveyi* DGC qrgB	Amp	[4]
**pCMW98**	pCMW75 with point mutation to inactivate	Amp	[4]

Table S2. PCR Primers used for this study

Primer	Sequence	Description
**CF101**	AGA TGA TGC TAG ATA TTA ATT TAG	Forward primer for *cbrR* with flanking region
**CF102**	GTT CGC TTG CAT TAA AGC AG	Reverse primer for *cbrR* with flanking region
**CF103**	ACC GGT CAT AGT GTG TAA AAT ACC TTT A	Inverse primer 1 to delete *cbrR* CDS from pCAF101 but retain flanking region to create pCAF102
**CF104**	ACC GGT TTT GCT AGC GCA AGT AAA ATA TTG TCT CAA G	Inverse primer 2 to delete *cbrR* CDS from pCAF101 but retain flanking region to create pCAF102
**CF105**	GCT AGC CAT TAT TCC CTC CAG GTA TTA TTT ATT CAG CAA GTC TT	Reverse primer to amplify the *rpsL*$_{HP}$-*cat* cassette from pKR021
***rpsL*$_{HP}$-F1**	ACC GGT AAC GAC TAA AGT TTT AAC A	Forward primer to amplify the *rpsL*$_{HP}$-*cat* cassette from pKR021
**CF110**	GGG TCT AGA TTA AAG AGC TTG CTA GGA TGG TAA GTG G	Forward primer to amplify *cbrR* CDS from pCAF101 and clone into pRRC to make pCAF108

**CF111**	GGG TCT AGA TTA AGA ACA TAC TTC AAC TCT ATC CTT GCC	Reverse primer to amplify *cbrR* CDS from pCAF101 and clone into pRRC to make pCAF108
**CF112**	GGG CAT ATG AAT AAG AAA ATT TTA ATT	Forward primer to amplify *cbrR* CDS from pCAF101 and clone into pET20b(+) to make pCAF107
**CF113**	GGG CTC GAG AGA ACA TAC TTC AAC TCT ATC	Reverse primer to amplify *cbrR* CDS from pCAF101 and clone into pET20b(+) to make pCAF107
**CF114**	ATA TAG TGC AGC AAA AAT TTG TAT TTT AC	Forward primer for SDM of pCAF107 to make the E334A substitution
**CF115**	CTA CCT ACA AGA TCT CTT C	Reverse primer for SDM of pCAF107 to make the E334A substitution
**CF116**	GCA GCT CTT GTA GGT AGA TAT AGT GC	Forward primer for SDM of pCAF107 to make the KGRD(323-326)AAAA substitution
**CF117**	TGC TGC AGT TTC ATT AAC TAT TTC ATT GG	Forward primer for SDM of pCAF107 to make the KGRD(323-326)AAAA substitution

1. Black RE, Levine MM, Clements ML, Hughes TP, Blaser MJ. Experimental Campylobacter jejuni infection in humans. J Infect Dis. 1988;157(3):472-9.

2. Hendrixson DR, Akerley BJ, DiRita VJ. Transposon mutagenesis of Campylobacter jejuni identifies a bipartite energy taxis system required for motility. Molecular microbiology. 2001;40(1):214-24.
3. Karlyshev AV, Wren BW. Development and application of an insertional system for gene delivery and expression in Campylobacter jejuni. Appl Environ Microbiol. 2005;71(7):4004-13.
4. Waters CM, Lu W, Rabinowitz JD, Bassler BL. Quorum sensing controls biofilm formation in Vibrio cholerae through modulation of cyclic di-GMP levels and repression of vpsT. Journal of bacteriology. 2008;190(7):2527-36.
